# Characteristics and outcomes of patients undergoing anesthesia while SARS-CoV-2 infected or suspected: a multicenter register of consecutive patients

**DOI:** 10.1186/s12871-022-01581-0

**Published:** 2022-02-14

**Authors:** Arthur James, Audrey De Jong, Thomas Jeanmougin, Antonia Blanie, Samy Figueiredo, Pierre Goffin, Morgan Le Guen, Elie Kantor, Flora Cipriani, Sébastien Campion, Mathieu Raux, Samir Jaber, Emmanuel Futier, Jean-Michel Constantin, For the Société Française d’Anesthésie Réanimation (SFAR) Research Network, Gael De Rocquigny, Agnes Le Gouez, Valentin Lefrançois, Safia Zioui, Jules Greze, Eleni Pagoni, Floriane Puel, Carole Buisset, Raphael Cinotti, Christophe Péricard, Adrien Lemoine, Jean Luc Soubirou, Mathieu Fontaine

**Affiliations:** 1 Département d’Anesthésie Réanimation, Sorbonne Université, GRC 29, AP-HP, DMU DREAM, Groupe Hospitalier Universitaire APHP-Sorbonne Université, Site Pitié-Salpêtrière, 75013 Paris, France; 2 Department of Anesthesia and Intensive Care Unit, Regional University Hospital of Montpellier, St-Eloi Hospital, University of Montpellier, PhyMedExp, INSERM U1046, CNRS UMR, 9214CEDEX 5 Montpellier, France; 3 Department of Anesthesiology and Intensive Care Unit, Kremlin Bicêtre, France; 4 Department of Anesthesiology and Critical Care, Groupe Santé CHC, MontLegia Hospital, Liège, Belgium; 5 Department of Anesthesiology and Pain Medicine, University of Versailles Saint Quentin, Hôpital Foch, Suresnes, France; 6 Department of Anesthesia and Surgical Critical Care, DMU PARABOL, Hôpital Bichat Claude Bernard, AP-HP Paris, France; 7 Department of Anesthesiology and Critical Care, Hôpital Beaujon, Clichy, France; 8 Département d’Anesthésie Réanimation, INSERM, UMRS1158 Neurophysiologie Respiratoire Expérimentale Et Clinique, AP-HP, Groupe Hospitalier Universitaire APHP-Sorbonne Université, Site Pitié-Salpêtrière, Sorbonne Université, 75013 Paris, France; 9 Département de Médecine Périopératoire, Anesthésie Et Réanimation, Centre Hospitalier Universitaire Clermont-Ferrand, Clermont-Ferrand, France; 10 GReD; UMR/CNRS6293; INSERM U1103, Université Clermont Auvergne, Clermont-Ferrand, France

**Keywords:** SARS-CoV-2, Respiratory complications, Infection, Perioperative care, Ventilation

## Abstract

**Background:**

There are limited data to detail the perioperative anesthetic management and the incidence of postoperative respiratory complications among patients requiring an anesthetic procedure while being SARS-CoV-2 positive or suspected.

**Methods:**

An observational multicenter cohort study was performed including consecutive patients who were SARS-CoV-2 confirmed or suspected and who underwent scheduled and emergency anesthesia between March 17 and May 26, 2020.

**Results:**

A total of 187 patients underwent anesthesia with SARS-CoV-2 confirmed or suspected, with ultimately 135 (72.2%) patients positive and 52 (27.8%) negative. The median SOFA score was 2 [0; 5], and the median ARISCAT score was 49 [36; 67]. The major respiratory complications rate was 48.7% (*n* = 91) with 40.4% (*n* = 21) and 51.9% (*n* = 70) in the SARS-CoV-2–negative and –positive groups, respectively (*p* = 0.21). Among both positive and negative groups, patients with a high ARISCAT risk score (> 44) had a higher risk of presenting major respiratory complications (*p* < 0.01 and *p* = 0.1, respectively).

**Discussion:**

When comparing SARS-COV-2–positive and –negative patients, no significant difference was found regarding the rate of postoperative complications, while baseline characteristics strongly impact these outcomes. This finding suggests that patients should be scheduled for anesthetic procedures based on their overall risk of postoperative complication, and not just based on their SARS-CoV-2 status.

**Supplementary Information:**

The online version contains supplementary material available at 10.1186/s12871-022-01581-0.

## Introduction

While the SARS-CoV-2 epidemic continues spreading around the world, and some countries already face a third or fourth wave, more and more patients will require anesthesia while being SARS-CoV-2 positive for either emergency or scheduled procedures that cannot be postponed. As a result, many caregivers will remain involved in the perioperative management of SARS-CoV-2–positive patients.

However, general anesthesia, particularly when associated with intubation and mechanical ventilation, is a situation that involves a risk of postoperative pulmonary complications [1]. This risk needs to be particularly acknowledged in the current pandemic context. SARS-CoV-2 has indeed been reported to induce an intense systemic inflammation response [2, 3] with a preferential pulmonary tropism [4, 5]. The pulmonary vulnerability of SARS-CoV-2–positive patients is therefore likely to have a major impact on postoperative outcomes, and especially on early pulmonary complications [6].

When the pandemic struck, the containment resulted in the cessation of all nonurgent hospital activities, while the surge of intensive care unit (ICU) patients led to the immediate transfer of operating rooms’ medical and paramedical staff to newly opened ICUs. While a fourth or even fifth peak is now a reality in many countries, and while surgical activity needs to be maintained and protected as much as possible, several issues are arise. One of them is the need to define structured health-care pathways for SARS-CoV-2 patients who require an anesthesia procedure while or after being symptomatic. To answer this question, stakeholders need to be able, for each patient, to have an accurate understanding of the risk–benefit balance with, on one hand, a potential risk of pulmonary complications and, on the other hand, the risk in postponing carcinologic, vascular, cardiac, or neurosurgical procedures, with a consequent significant morbidity [7]. This understanding will allow physicians to propose an informed decision-making and a truthful informing of patients, and to anticipate the required resources for the optimal care of their patients. In the literature, several publications detailing the perioperative management of SARS-CoV-2 patients are available, but mostly focus on surgical management and outcomes or report a small collective of patients [8–11].

The first main aim of this study was to compare the incidence of major respiratory complications between SARS-CoV-2–positive and –negative patients. The second main aim was to describe preoperative conditions and perioperative management of SARS-CoV-2 patients.MateriAl and methodS.

This observational multicenter cohort study used medical data collected anonymously. To avoid selection bias, all centers were committed to include consecutive patients only. Each center included patients between March 17 and May 26, 2020. In this study, 19 centers in France and Belgium participated. This report follows the STROBE statement for the reporting of observational studies (Supplementary material 1) [12].

### Ethics

Ethical approval for this study (IRB 00,010,254–2020-049) was provided by the Ethical Committee of Société Francaise d’Anesthésie Réanimation, Paris, France (Chairperson Prof. J.E. Bazin) on March 31, 2020. This study has been registered in the *Registre des traitements de l’Assistance Publique des Hôpitaux de Paris*, n°20,200,716,194,220. Informed consent was obtained from all participants.

### Study population

Non-obstetrical adult patients undergoing scheduled or emergency procedures were included if they required anesthesia while being SARS-CoV-2 positive or suspect at the time of the inclusion. Anesthesia could be undertaken for multiple reasons, including surgery, endoscopic, or interventional radiologic procedures. Ventilated patients who underwent scheduled surgical tracheotomy were excluded, since they were all symptomatic for more than 3 weeks and were already ventilated in an intensive care unit at the time of the surgery.

At their arrival, patients were classified as positive cases if the suspicion was confirmed with laboratory testing based on viral RNA detection by quantitative RT-PCR. Patients were classified as suspect cases if they were not yet confirmed but a physician had decided to treat them as if they were positive until a confirmation was obtained. All patients were retrospectively categorized (positive or negative) based on their RT-PCR SARS-CoV-2 status on the preoperative nasopharyngeal swab.

Both positive and suspected cases received the same immediate postoperative management, including recovery in the operating room and dedicated ICU rooms, depending on the center of inclusion, and the same prescriptions and follow-up.

Patients without SARS-COV-2 status confirmation during their hospital stay were also excluded. If a single patient underwent multiple consecutive anesthetic procedures, we included the first procedure.

### Study outcomes

We used as the main outcome the proportion of patients having a major respiratory complication up to 7 days (re-intubation or unexpected noninvasive ventilation requirement or unexpected high-flow oxygen therapy during the first 6 h, respiratory failure up to 7 days, or pneumonia up to 7 days) [13]. Respiratory failure was defined as a SpO_2_ < 92% [14], the need for more than 3 L of oxygen per minute, or mechanical ventilation with a FiO_2_ > 0.6. Pneumonia was defined as the decision of a physician to treat a pulmonary infection with antibiotics.

Secondary outcomes were the proportion of patients discharged alive up to both 7 and 28 days, in-hospital mortality up to both 7 and 28 days, the proportion of patients having an AKIN score ≥ 2 up to 7 days [15], and the proportion of patients who required ICU admission immediately after surgery.

### Data collection

Descriptive data of SARS-CoV-2 symptoms (fever, signs of pneumonia, date of the first symptoms), of surgery (type of surgery, emergency, length of stay in operating room) as well as patients’ baseline characteristics (American Society of Anesthesiology physical score [ASA-PS], treatments, history of hypertension, diabetes or obesity), scores (Sequential Organ Failure Assessment [SOFA] [16], ARISCAT risk groups [1], and Surgery Risk Stratification [17]) and biology at admission (serum creatinine, hemoglobin, leukocytes, lymphocytes) were collected. Therapeutic measures implemented in the operating room (type of anesthesia, ventilation mode, main ventilatory parameters, unexpected requirement of a recruitment maneuver, transfusion, use of vasoactive drugs, locoregional anesthesia), especially those relative to SARS-CoV-2 guidelines (rapid sequence induction, use of a closed loop aspiration system, use of a video laryngoscope), were also collected [18]. We also collected oxygen support and treatments required during the first 6 h after the surgery (noninvasive ventilation, high-flow oxygen therapy, emergency intubation). The SpO_2_/FiO_2_ ratio was used as an oxygenation parameter [19]. The FiO_2_ was calculated for non-intubated patients based on the oxygen flow (L/min) delivered with nasal cannula or face mask (see correspondence in Supplementary material 2) [20].

During the first 7 days after surgery, local investigators used medical and administrative records to identify major medical events (intensive care admission, pneumonia, acute kidney injury), the proportion of patients still in hospital, and 7 days’ all-cause mortality. We also evaluated 28 days’ all-cause mortality and the proportion of patients still being hospitalized at day 28.

### Data analysis

First, a descriptive analysis was performed using number and percentage for qualitative variables and median and interquartile range (IQR) for quantitative variables.

Second, patients confirmed as SARS-CoV-2 positive (“SARS-CoV-2–positive groups”) were compared to those who were finally ruled out from the diagnosis (“SARS-CoV-2–negative groups”). All data were censored on day 28 after the procedure. Comparisons between groups were performed using the Mann–Whitney test or Fisher test when adapted. All comparisons were two-tailed.

Third, we conducted a subgroup analysis to explore, among the SARS-CoV-2–positive and –negative patients, the impact of the preoperative risk of major respiratory complications based on the ARISCAT risk groups (with ARISCAT < 26 being considered as low risk, 26–44 as intermediate risk, and > 44 as high risk) [1].

Missing data were reported for each variable. No imputation was made except for the primary outcome, as the lack of reporting of complications was classified as “no complication.” A sensitivity analysis was then performed, excluding these patients with a lack of reporting of complications.

To address multiplicity, *p* values were not calculated to describe the study population, and using the Bonferroni method, we considered a *p* value < 0.001 for significance. All analyses were conducted with R v.4.0.2 (http://www.R-project.org).

## Results

### Descriptive analysis

A total of 200 adult patients were consecutively included. Each center included between 1 and 51 patients. Among them, 13 were secondarily excluded, meeting the exclusion criteria (tracheotomy, *n* = 8; unknown SARS-CoV-2 status at discharge, *n* = 5) (Supplementary material 3). A total of 187 patients who underwent an anesthesia while being SARS-CoV-2 positive or suspect were analyzed. Among these patients, 111 (59.4%) were SARS-CoV-2 confirmed as being SARS-CoV-2 positive at the time of the inclusion, while 76 (40.6%) were still considered as suspected. Patients included while SARS-CoV-2 suspected were secondarily either confirmed as positive (*n* = 24/76, 31.6%) or not (*n* = 52/76, 68.4%). The included patients were categorized into two groups: those who were finally confirmed as SARS-CoV-2 positive (*n* = 135, 72.2%) and those who were negative as SARS-CoV-2 (*n* = 52, 27.8%) (Supplementary material 4).

The median population age was 65 years [54; 76], with 118 patients (63%) being men. The median delay between the first symptoms and the anesthesia procedure was 12 days [4; 27]. At the time of the surgery, 83 patients (44.9%) had respiratory symptoms of pneumonia.

Among all procedures, 65 (34.7%) were carried out oncall time. Patients underwent several types of surgery, with visceral surgery (*n* = 48; 25.7%), orthopedic surgery (*n* = 41; 21.9%), urologic surgery (*n* = 18; 9.6%), vascular surgery (*n* = 20; 10.7%), gastrointestinal procedures (*n* = 16; 8.6%), neurosurgery (*n* = 19; 10.2%), and other types of surgery (*n* = 25; 13.3%).

Patients with significant comorbidities were included, with 114 (61%) having an ASA-PS superior or equal to 3, a median SOFA score of 2 [0; 5], and, respectively, 107 (57.2%) and 63 (33.7%) being classified as high or intermediate risk on the ARISCAT score.

Before the anesthesia procedure, 39 (23.0%) were coming from the emergency department, 49 (28.8%) were hospitalized in the medical ward, 24 (14.1%) in the surgical ward, 37 (21.8%) in the intensive care unit, and 21 (12.4%) were transferred directly from another hospital. Almost half of the patients required oxygen support, whether with conventional oxygen therapy (*n* = 47, 25.5%) or mechanical ventilation (*n* = 37, 20.1%). These results are summarized in Table 1.

**Table 1:** Baseline characteristics

	**Population (*n* = 187)**	**SARS-CoV-2 negative (*n* = 52)**	**SARS-CoV-2 positive (*n* = 135)**
Teaching hospital	137 (73.3)	40 (76.9)	97 (71.9)
Patients origin			
Emergency department	39 (23.0)	16 (35.6)	23 (17.6)
Surgical ward	24 (14.1)	3 (6.7)	21 (16.8)
Medical ward	49 (28.8)	13 (28.9)	36 (28.8)
Intensive care unit	37 (21.8)	4 (8.9)	33 (26.4)
Transfer	21 (12.4)	9 (20.0)	12 (9.6)
Age (years)	65 [54; 76]	69 [55; 77]	64 [53; 76]
Gender, male	118 (63.4)	30 (57.7)	88 (65.7)
Delay first symptoms—anesthesia	12 [4; 27]	3 [2; 9]	17 [8; 31]
Delay first symptoms – anesthesia			
First week	49 (33.1)	26 (66.7)	23 (21.1)
Second week	29 (19.6)	7 (17.9)	22 (20.2)
Third week	25 (16.9)	1 (2.6)	24 (22.0)
Fourth week	8 (5.4)	0 (0.0)	8 (7.3)
Fith week and more	37 (25.0)	5 (12.8)	32 (29.4)
Respiratory symptoms	83 (44.9)	13 (25.5)	70 (52.2)
Surgical specialty			
Visceral	45 (25.7)	19 (36.5)	29 (21.5)
Orthopedic	41 (21.9)	6 (11.5)	35 (25.9)
Urology	18 (9.6)	7 (13.5)	11 (8.1)
Vascular	20 (10.7)	9 (17.3)	11 (8.1)
Gastro Intestinal endoscopy	16 (8.6)	6 (11.5)	10 (7.4)
Neurosurgery	19 (10.2)	3 (5.8)	16 (11.9)
Other	25 (13.3)	2 (3.8)	23 (17.0)
Emergency surgery	163 (87.6)	47 (90.4)	116 (86.6)
Surgical delay for emergency patients, min	360 [177; 1380]	240 [121; 720]	360 [180; 1440]
Surgery Risk Stratification			
Very low risk	15 (8.0)	5 (9.6)	10 (7.4)
Low risk	38 (20.3)	8 (15.7)	30 (22.2)
Intermediate risk	98 (52.4)	25 (48.1)	73 (54.1)
High risk	31 (16.6)	12 (23.1)	19 (14.1)
Very high risk	5 (2.7)	2 (3.8)	3 (2.2)
ARISCAT Score	49 [36; 67]	40 [27; 48]	56 [41; 68]
ARISCAT Risk Group, %			
Low	17 (9.1)	11 (21.2)	6 (4.4)
Intermediate	63 (33.7)	22 (42.3)	41 (30.4)
High	107 (57.2)	19 (30.5)	88 (65.2)
Active smoking	27 (14.5)	11 (21.2)	16 (11.9)
ASA-PS			
1	16 (8.6)	4 (7.7)	12 (9.0)
2	56 (30.1)	15 (28.8)	41 (30.6)
3	81 (43.5)	26 (50.0)	55 (41.0)
4	33 (17.4)	7 (13.5)	26 (19.4)
High Blood pressure	93 (50.3)	24 (46.2)	69 (51.9)
BMI, kg.m^–2^	25.6 [23; 29]	25 [22; 29]	26 [23; 29]
BMI > = 30	39 (22.3)	11 (22.9)	28 (22.0)
Current treatment			
Immunosuppressors	14 (7.5)	2 (3.8)	12 (9.0)
Steroids	22 (11.8)	4 (7.7)	18 (13.4)
Antihypertensives	50 (27.0)	14 (26.9)	36 (27.1)
Oral antidiabetics	23 (12.4)	5 (9.6)	18 (13.4)
NSAIDS	8 (4.3)	4 (7.7)	4 (3.0)
Insulin	28 (15.1)	4 (7.7)	24 (18.0)
Radiotherapy or chemotherapy	11 (7.1)	3 (7.0)	8 (7.1)
SOFA Score	2 [0; 5]	2 [1; 5]	2 [0; 5]
Creatinine, mmol/L	80 [59; 120]	87 [65; 123]	79 [56; 115]
Hemoglobin, g/dL	11.4 [9.6; 13.3]	12.6 [10.9; 13.9]	11.0 [8.7; 13.0]
White blood cell count, × 10^9^/L	10,5 [7.0; 15.2]	12.3 [7.9; 17.9]	10.0 [6.8; 14.3]
Lymphocyte count, × 10^9^/L	1.0 [0.7; 1.6]	1.0 [0.8; 1.8]	1.1 [0.7; 1.4]
Preoperative SpO2, %	97 [95; 99]	96 [95; 99]	97 [95; 99]
SpO2/FiO2 ratio	440 [250; 462]	448 [345; 462]	419 [213; 462]
Preoperative oxygen support			
No oxygen	100 (54.3)	33 (64.7)	67 (50.4)
Oxygen ≥ 1L.min^−1^	47 (25.5)	14 (27.5)	33 (24.8)
Mechanical ventilation	37 (20.1)	4 (7.8)	33 (24.8)

Data are median [interquartile range] and No./Total (%)

Other types of surgery include cardiac (*n* = 2, 1.1%), gynecologic surgery (*n* = 1, 0.5%), ear, nose and throat surgery (*n* = 7, 3.7%). thoracic surgery (*n* = 8, 4.3%) and interventional radiology (*n* = 7, 3.7%)

Pre-operative oxygen requirement was defined as: a SpO2 < 92% or the need of more than 3L of oxygen per minute or mechanical ventilation with a FiO2 > 0.6

*ASA-PS *American Society of Anaesthesiology physical score, *BMI *Body Mass Index, *NSAIDS *Non Steroid And Anti-Inflammatory DrugS, *SpO2 *peripheral oxygen saturation, *SOFA *Sequential Organ Failure Assessment

Procedures were mostly performed under general anesthesia (*n* = 173, 93.5%), and 26 (14.8%) implied a locoregional anesthesia. A total of 164 procedures (88.2%) required mechanical ventilation, and 57 (33.1%) were performed using total intravenous anesthesia. Median surgery length was 70 min [37; 114].

Patients under mechanical ventilation had a median tidal volume of 6 mL/kg of ideal body weight [6;7], with a median positive end-expiratory pressure of 6 cmH_2_O [5; 7], and a median peak pressure of 21 cmH_2_0 [18;26]. Some patients required unplanned recruitment maneuver (*n* = 16, 10.6%), intraoperative transfusion (*n* = 21, 12.0%), or vasoactive drugs (*n* = 79, 43.9%). These results are summarized in Table 2.

**Table 2:** Perioperative management

	**Population (*n* = 187)**	**SARS-CoV-2 negative (*n* = 52)**	**SARS-CoV-2 positive (*n* = 135)**
General anesthesia	173 (93.5)	52 (100.0)	121 (91.0)
Rapid Sequence Induction	113 (86.3)	38 (90.5)	75 (84.3)
Videolaryngoscopy	110 (87.3)	33 (86.6)	77 (87.5)
Closed system for endotracheal suction	28 (37.3)	8 (28.6)	20 (42.6)
Total Intra-Venous Anesthesia	57 (33.1)	17 ( 33.3)	40 ( 33.0)
Mechanical ventilation	164 (88.2)	46 (88.5)	118 (88.1)
Mechanical ventilation characteristics			
Tidal volume, ml.kg^−1^ PBW	6 [6; 7]	7 [6; 7]	6 [6; 7]
PEEP, cmH2O	6 [5; 8]	5 [5; 6]	6 [5; 8]
FiO2, %	50 [44; 60]	59 [46; 60]	50 [43; 64]
Peak pressure, cmH2O	22 [18; 26]	20 [18; 23]	22 [18; 27]
Unplanned recruitment maneuver	16 (10.6)	3 (6.7)	13 (12.3)
Intra-operative transfusion	22 (12.0)	8 (15.4)	14 (10.7)
Totally intravenous fluids, mL	1000 [500; 1600]	1100 [500; 2000]	1000 [500; 1536]
Need of vasoactive drugs	79 (43.9)	25 (49.0)	54 (41.9)
Loco-Regional anesthesia	25 (14.5)	5 (9.6)	21 (16.9)
Surgery length, min	70 [37; 114]	60 [26; 104]	70 [40; 120]
Surgery length, min			
[0; 30]	40 (27.0)	17 (35.4)	23 (23.0)
[30; 60]	45 (30.4)	11 (22.9)	34 (34.0)
[60; 120]	29 (19.6)	10 (20.8)	19 (19.0)
[120; 540]	34 (23.0)	10 (20.8)	24 (24.0)

Data are median [interquartile range] and No./Total (%)

*PEEP* Positive end-expiratory pressure, *FiO2* Fraction of inspired oxygen, *PBW* Per Body Weight

Comparison between positive and negative SARS-CoV-2 patients.

The cumulative incidence of major respiratory complications based on the composite outcome was 91 (48.7%), with 21 (40.4%) and 70 (51.9%) in the SARS-CoV-2–negative and –positive groups, respectively (*p* = 0.21). Among these complications, the most frequent was the postoperative oxygen requirement (*n* = 51, 28.5%), followed by the postoperative medical treatment of a lower pulmonary tract infection (*n* = 39, 21.8%). Unexpected requirement of noninvasive ventilation (*n* = 3, 2.2%), high-flow oxygen (*n* = 1, 0.8%), or intubation (*n* = 0, 0%) were rare. Sensitivity analysis involving complete cases did not change these results (Supplementary Material 5).

Seven days’ in-hospital mortality was 15 (8.1%) with, respectively, 3 (5.8%) and 12 (9.0%) in the SARS-CoV-2–negative and –positive groups (*p* = 0.68). Twenty-eight days’ in-hospital mortality was 18 (13.1%), with 3 (8.1%) and 15 (15.0%) in the SARS-CoV-2–negative and –positive groups, respectively (*p* = 0.43).

The proportion of patients discharged alive at day 7 was 52 (28.1%), with 21 (40.4%) and 31 (23.3%) in the SARS-CoV-2–negative and –positive groups, respectively (*p* = 0.03). The proportion of patients discharged alive at day 28 was 94 (64.8%), with 32 (76.2%) and 62 (60.2%) in the SARS-CoV-2–negative and –positive groups, respectively (*p* = 0.10).

Independently of the SARS-CoV-2 status, half of the population required intensive care unit care immediately after anesthesia, whether because they were in intensive care before surgery (*n* = 36, 19.7%) or because their clinical status required an ICU admission after surgery (*n* = 41, 22.4%).

These results are summarized in Table 3.

**Table 3:** Study outcomes

	**Population (*n* = 187)**	**SARS-CoV-2 negative (*n* = 52)**	**SARS-CoV-2 positive (*n* = 135)**	***P*-value**
Major respiratory complications	91 (48.7)	21 (40.4)	70 (51.9)	0.21
Post-operative O2 requirement	51 (28.5)	9 (18.4)	42 (32.3)	0.10
Curative NIV before h6	3 (2.2)	0 (0.0)	3 (2.9)	
HFO before h6	1 (0.8)	0 (0.0)	1 (1.1)	0.66
Unplanned reintubation before h6	0 (0.0)	0 (0.0)	0 (0.0)	1.00
Pneumonia up to d7	39 (21.8)	9 (17.3)	30 (23.6)	0.47
ICU admission				
Immediately after surgery	41 (22.4)	17 (33.3)	24 (18.2)	0.02
Up to 7-day	67 (35.8)	19 (36.5)	48 (35.6)	1.00
AKI, AKIN				
Class ≥ 2	24 (13.8)	6 (12.2)	18 (14.5)	0.45
Discharge alive				
Up to 7-day	52 (28.1)	21 (40.4)	31 (23.3)	0.03
Up to 28- days	94 (64.8)	32 (76.2)	62 (60.2)	0.10
In-hospital death				
Up to 7-day	15 (8.1)	3 (5.8)	12 (9.0)	0.68
Up to 28- days	18 (13.1)	3 (8.1)	15 (15.0)	0.44

Data are median [interquartile range] and number (percentage)

*NIV* Non-invasive ventilation, *AKI* Acute kidney injury, *HFO* High flow oxygen therapy, *ICU* Intensive Care Units

### Risk stratification based on the ARISCAT score

**Fig. 1:**
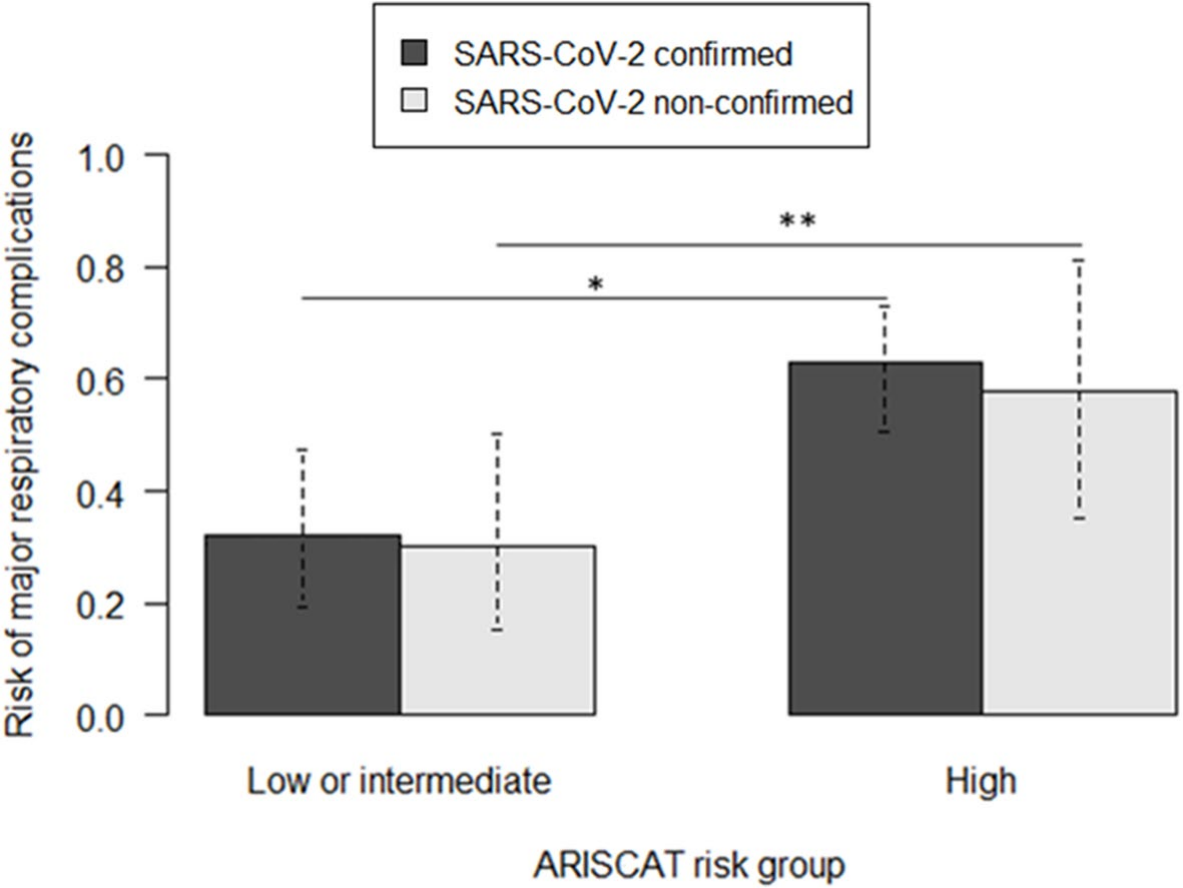
Risk of major respiratory complications according to the ARISCAT risk groups. ARISCAT risk groups are as follows: < 26, low risk; 26–44, intermediate risk; and > 44, high risk

Figure 1 illustrates that, in both SARS-CoV-2–positive and –negative groups, a high ARISCAT risk score implies more frequent major respiratory complications with:

1. in the SARS-CoV-2–positive group, 32% (*n* = 15/47, 95% *CI* [20–47]) in the low- or intermediate-risk subgroup, and 63% (*n* = 55/88, 95% *CI* [52–73]) in the high-risk subgroup (*p* < 0.01),
2. in the SARS-CoV-2–negative group, 30% (*n* = 10/33, 95% *CI* [16–49]) in the low- or intermediate-risk subgroup, and 58% (*n* = 11/19, 95% *CI*) in the high-risk subgroup (*p* = 0.10).

## Discussion

### Synthesis and interpretation

In this observational multicenter prospective study, we observed that patients requiring anesthesia while being SARS-CoV-2 positive or suspect at the time of the inclusion were mostly critical patients with several comorbidities, and they were ventilated according to recommendations in operating rooms. When comparing positive and negative SARS-CoV-2 patients, no significant difference was found regarding the postoperative cumulative incidence of major respiratory complications.

Despite the number of patients infected by SARS-CoV-2, there are still limited data on their perioperative management, and particularly on their ventilatory management and postoperative risk of complications. This information would be, however, important to have when the pandemic strikes again and leads more and more patients to require anesthesia, whether for emergency or scheduled procedures.

We did not identify an increased risk of major respiratory complications among SARS-CoV-2–positive patients. One explanation could be that, in a lowly hypoxemic preoperative population (median SpO_2_/FiO_2_ is 440 [250; 462]), the postoperative risk of pulmonary complications is strongly sustained by patients’ preoperative medical conditions (ASA-PS, ARISCAT, and SOFA scores) and surgical risk (emergency surgery, Surgery Risk Stratification, procedure length), which makes it difficult to isolate an effect of the SARS-CoV-2.

This study also warns health-care stakeholders that these patients are at high risk of postoperative complications, with an overall 43% incidence, and that 41% of this population requires intensive care after surgery.

### Literature

Few studies have been published addressing SARS-CoV-2 surgical patients, and most of them highlighted a significant increase in postoperative complications. In one of the first surgical cohorts published, Lei et al. focused on 34 patients who underwent scheduled surgery and developed symptoms only after the surgery [6]. In a population that involves mainly high-surgical-risk patients, they highlighted that, although asymptomatic before surgery, 44.1% of these patients required postoperative intensive care, with a mortality rate of 20.5%. A case–control study published recently and involving 41 SARS-CoV-2–positive patients found these patients to be at higher risk of mortality, respiratory complications, or thrombosis when matched to non-SARS-CoV-2 patients, but with a wide confidence interval suggesting an important heterogeneity [9]. The most important published study included 1,128 patients and reported a 30-day mortality rate of 23.8% with risk factors such as being a man, older than 70 years, with an ASA-PS greater or equal to 3, and emergency major surgery [8]. However, no control group was used, not allowing the comparison between patients with and without SARS-CoV-2. All these studies were conducted from a surgical perspective, with minimal information on perioperative ventilation parameters, which are, however, known to be a strong determinant of postoperative pulmonary outcomes [21].

When the epidemic broke out, guidelines for perioperative management of SARS-CoV-2–positive patients were published [18, 22, 23]. A high compliance with these guidelines was highlighted in this study with, respectively, 86.3% (*n* = 113) and 87.3% (*n* = 110) of the inductions that implied rapid sequence induction and videolaryngoscopy use, even for inductions that did not require such methods. We also highlighted that anesthetists seemed to have a strong awareness of the importance of perioperative protective ventilation parameters, with a median low tidal volume of 6 [6; 7] mL/kg PBW and a median PEEP of 6 [6; 7] cmH_2_O.

A significant proportion of the patients included in this study were ultimately found not to be infected by SARS-CoV-2. Nevertheless, we considered it relevant to include them for several reasons: 1) their pre-, per-, and immediate postoperative management were similar to SARS-CoV-2–positive patients, who constitute a meaningful control group; 2) their inclusion reflects a real burden of work for anesthesiologists involved in the management of SARS-CoV-2 patients; and 3) SARS-CoV-2 acute respiratory distress syndrome has been discussed to be similar to other causes of ARDS [24].

### Weaknesses

#### This study has several weaknesses

First, it included consecutive patients with different types of procedures who were recruited in 19 different centers. This strategy, with an important number of centers compared to the small number of patients included, might have increased heterogeneity among included patients and health-care strategies. Nevertheless, it allows capturing a broad picture closest to real life and prevents selection bias.

Second, our SARS-CoV-2 testing strategy was mainly based on nasopharyngeal RT-PCR, which has been reported to suffer from a high false-negative rate [25], a situation that could have placed patients in the SARS-CoV-2–negative group who were actually infected. This problem has been addressed in most hospitals by doing repeated RT-PCR in equivocal clinical situations and using CT scans when useful.

Third, we included patients with a broad interval of delay between the first symptoms and anesthesia, which suggests that some patients who were included might be at the most active phase of the infection, while others were at the beginning of the recovery phase. This heterogeneity is likely to have affected this study’s capacity to identify a significant difference between SARS-CoV-2–positive and –negative patients.

Fourth, among patients included while SARS-CoV-2 was suspected, 52 were finally confirmed as SARS-CoV-2 negative. They, however, had evocative symptoms that led to the suspicion, and this study was not designed to report the final respiratory diagnosis. Nevertheless, with 87.6% of patients requiring emergency surgery and 21.8% coming from ICU, we hypothesize that these respiratory symptoms are likely to be related to patients’ inflammatory status and various other lung injuries, which are responsible for a high rate of pulmonary complications.

Fifth, the incidence of respiratory complications is much higher in the study population (whatever SARS-CoV-2 status) than would be expected based on the assessment of ARISCAT. Such a situation has already been observed with a higher risk of postoperative ARDS after scheduled cardiac surgery during flu epidemic months [26]. It also illustrates that this study control group is composed of a respiratory symptomatic population with frequent severe underlying conditions.

Finally, we conducted a prospective cohort study in a limited period of time including 187 patients, which allows a limited power. Recruiting more patients would possibly allow identifying statistically significant differences between the two groups; however, regardless of the number of patients included, it would remain extremely difficult to account for the individual effect of the SARS-CoV-2 disease and for the surgical risk of complications in each group.

### Implications

Patients requiring an anesthesia while being SARS-CoV-2 positive or suspect at the time of the inclusion need to be considered during their preoperative evaluation as being at high risk to present postoperative complications and to develop severe forms of the disease, according to the WHO clinical progression scale [27]. These complications might be directly related to the systemic SARS-CoV-2 impact, but might also be related to patient preoperative health status or to the surgical condition involved.

Considering this high risk of postoperative complications for SARS-CoV-2 patients and the risk of contaminating other patients and caregivers, scheduled surgery obviously needs to be postponed. When postponement is not possible (urgency or emergency), the assessment should encompass the SARS-CoV-2 status and the global risk. We thus advocate that these patients need to be preoperatively carefully screened to stratify perioperative health-care strategies on patient individual risk. For this screening, following the old saying *we fight like we train* [28], the use of scores that are used daily for a long time seems to be relevant.

## Conclusion

Among patients requiring an anesthesia while being SARS-CoV-2 positive or suspect at the time of the at the time of the inclusion in the study, no significant difference in outcomes was found when comparing those who were confirmed SARS-CoV-2 positive and those who ultimately tested negative for SARS-CoV-2, while patients’ baseline characteristics strongly influenced their outcomes. This situation implies that patients requiring urgent and non-postponable surgery need to be evaluated not only based on the SARS-CoV-2 status, but also according to an overall evaluation of the perioperative risk, including patient preoperative health status and surgical requirements.

## Supplementary Information

**Additional file 1.** Supplementary material.

## Data Availability

Data can be available after request to the corresponding author and discussion with the working group.
