# Supplementary material for: Characteristics and outcomes of patients undergoing anesthesia while SARS-CoV-2 infected or suspected: a multicenter register of consecutive patients
Source: BMC Anesthesiol. 2022 Feb 14;22:46. doi: 10.1186/s12871-022-01581-0 (PMC8842968; doi:10.1186/s12871-022-01581-0)
Supplement: Supplementary file 1 — Additional file 1. Supplementary material. [file 12871_2022_1581_MOESM1_ESM.docx]

# Supplementary material

## Supplementary material 1: STROBE Check-list

|  | Item No | Recommendation | Page |
| --- | --- | --- | --- |
| Title and abstract | 1 | (*a*) Indicate the study’s design with a commonly used term in the title or the abstract | 1&3 |
|  |  | (*b*) Provide in the abstract an informative and balanced summary of what was done and what was found | 3 |
| Introduction | | |  |
| Background/rationale | 2 | Explain the scientific background and rationale for the investigation being reported | 5 |
| Objectives | 3 | State specific objectives, including any prespecified hypotheses | 6 |
| Methods | | |  |
| Study design | 4 | Present key elements of study design early in the paper | 7 |
| Setting | 5 | Describe the setting, locations, and relevant dates, including periods of recruitment, exposure, follow-up, and data collection | 7 |
| Participants | 6 | (*a*) Give the eligibility criteria, and the sources and methods of selection of participants. Describe methods of follow-up | 8 |
|  |  | (*b*) For matched studies, give matching criteria and number of exposed and unexposed | NA |
| Variables | 7 | Clearly define all outcomes, exposures, predictors, potential confounders, and effect modifiers. Give diagnostic criteria, if applicable | 8 |
| Data sources/ measurement | 8* | For each variable of interest, give sources of data and details of methods of assessment (measurement). Describe comparability of assessment methods if there is more than one group | 8 |
| Bias | 9 | Describe any efforts to address potential sources of bias | 7 |
| Study size | 10 | Explain how the study size was arrived at | NA |
| Quantitative variables | 11 | Explain how quantitative variables were handled in the analyses. If applicable, describe which groupings were chosen and why | 10 |
| Statistical methods | 12 | (*a*) Describe all statistical methods, including those used to control for confounding | 10 |
|  |  | (*b*) Describe any methods used to examine subgroups and interactions | 10 |
|  |  | (*c*) Explain how missing data were addressed | 10 |
|  |  | (*d*) If applicable, explain how loss to follow-up was addressed | 10 |
|  |  | (*e*) Describe any sensitivity analyses | 10 |
| Results | | |  |
| Participants | 13* | (a) Report numbers of individuals at each stage of study—eg numbers potentially eligible, examined for eligibility, confirmed eligible, included in the study, completing follow-up, and analysed | 11 |
|  |  | (b) Give reasons for non-participation at each stage | 11 |
|  |  | (c) Consider use of a flow diagram | Sup Mat |
| Descriptive data | 14* | (a) Give characteristics of study participants (eg demographic, clinical, social) and information on exposures and potential confounders | 11 |
|  |  | (b) Indicate number of participants with missing data for each variable of interest | Sup Mat |
|  |  | (c) Summarise follow-up time (eg, average and total amount) | 12 |
| Outcome data | 15* | Report numbers of outcome events or summary measures over time | 12 |
| Main results | 16 | (*a*) Give unadjusted estimates and, if applicable, confounder-adjusted estimates and their precision (eg, 95% confidence interval). Make clear which confounders were adjusted for and why they were included | 12 |
|  |  | (*b*) Report category boundaries when continuous variables were categorized | NA |
|  |  | (*c*) If relevant, consider translating estimates of relative risk into absolute risk for a meaningful time period | NA |
| Other analyses | 17 | Report other analyses done—eg analyses of subgroups and interactions, and sensitivity analyses | 12 |
| Discussion | | |  |
| Key results | 18 | Summarise key results with reference to study objectives | 15 |
| Limitations | 19 | Discuss limitations of the study, taking into account sources of potential bias or imprecision. Discuss both direction and magnitude of any potential bias | 17 |
| Interpretation | 20 | Give a cautious overall interpretation of results considering objectives, limitations, multiplicity of analyses, results from similar studies, and other relevant evidence | 15-16 |
| Generalisability | 21 | Discuss the generalisability (external validity) of the study results | 16 |
| Other information | | |  |
| Funding | 22 | Give the source of funding and the role of the funders for the present study and, if applicable, for the original study on which the present article is based | 21 |

## Supplementary material 2: FiO_2_ correspondence ^17^

| **Oxygen flow (L/mn)** | **Estimated FiO_2_** |
| --- | --- |
| 0 | 0.21 |
| 1 | 0.24 |
| 2 | 0.28 |
| 3 | 0.32 |
| 4 | 0.36 |
| 5 | 0.40 |
| 6 | 0.50 |
| 7 | 0.50 |
| 8 | 0.60 |
| 9 | 0.60 |
| 10 | 0.60 |

## Supplementary material 3: patients’ recruitment by centers

|  | Inclus |
| --- | --- |
| APHP – Hôpital de la Pitié-Salpétrière, Paris | 51 |
| APHP – Hôpital de Bicêtre, Le Kremlin-Bicêtre | 25 |
| Groupe Santé CHC, Hôpital MontLégia, Liège | 21 |
| Hôpital Foch, Suresnes | 19 |
| APHP - Hôpital Beaujon, Clichy | 10 |
| APHP - Hôpital Bichat, Paris | 10 |
| Hôpital de Rangueil, Toulouse | 9 |
| Hôpital Sainte Musse, Toulon | 7 |
| CHU de Caen, Caen | 6 |
| CH Saint-Joseph Saint-Luc, Lyon | 6 |
| Hôpital Antoine Béclère, Clamart | 5 |
| HIA – Begin, Saint-Mandé | 4 |
| APHP – Tenon, Paris | 4 |
| Hôpital Simone Veil, Eaubonne | 2 |
| CH Poitier, Poitier | 2 |
| Hôpital de Hautepierre, Strasbourg | 2 |
| CHU Grenoble, Grenoble | 1 |
| Centre Léon Bérard, Lyon | 1 |
| Hôpital Louis Pradel, Lyon | 1 |
| CHU Nantes, Nantes | 1 |

APHP, Assistance Publique des Hôpitaux de Paris ; CH, Centre Hospitalier ; CHU, Centre Hospitalier Universitaire

## Supplementary material 4: Study Flowchart


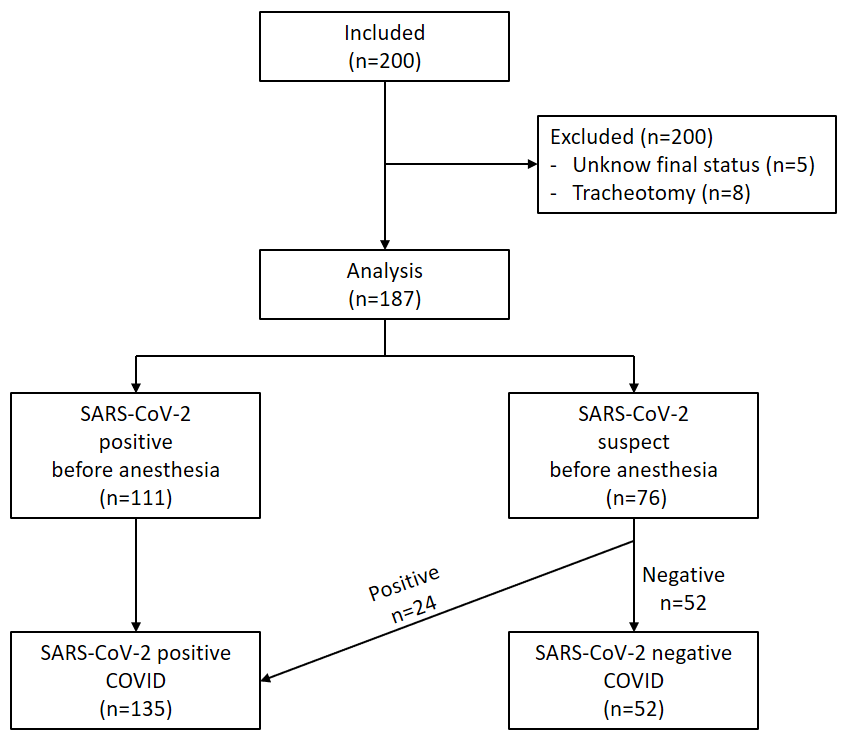


## Supplementary material 5: Sensitivity analysis: Complete cases analysis of the main outcome

|  | **Population**  **(n=121)** | **SARS-CoV-2 negative**  **(n=35)** | **SARS-CoV-2 positive**  **(n=86)** | **p-value** |
| --- | --- | --- | --- | --- |
| Major respiratory complications | 42 (34.7) | 10 (28.6) | 32 (37.2) | 0.49 |

Data are median [interquartile range] and number (percentage).

NIV, Non-invasive ventilation; AKI, acute kidney injury; HFO, high flow oxygen therapy, ICU, Intensive Care Units
